# Performance of the VITEK^®^ MS system for the identification of filamentous fungi in a microbiological laboratory in Chile

**DOI:** 10.1371/journal.pone.0315690

**Published:** 2024-12-23

**Authors:** Lorena Porte, Rodrigo Cruz, Inia Pérez, Carmen Varela, Cristina Díaz, Patricia García, Paulette Legarraga, Francisca Valdivieso, Thomas Weitzel

**Affiliations:** 1 Laboratorio Clínico, Clínica Alemana, Facultad de Medicina Clínica Alemana, Universidad del Desarrollo, Santiago, Chile; 2 Centro de Diagnóstico e Investigación de Enfermedades Infecciosas, Universidad de Valparaíso, Valparaíso, Chile; 3 Servicio de Infectología, Departamento de Medicina Interna, Clínica Alemana, Facultad de Medicina Clínica Alemana, Universidad del Desarrollo, Santiago, Chile; 4 Programa de Microbiología y Micología, ICBM, Facultad de Medicina, Universidad de Chile, Santiago, Chile; 5 Departamento de Laboratorios Clínicos, Escuela de Medicina, Pontificia Universidad Católica de Chile, Santiago, Chile; 6 Laboratorio de Microbiología, Hospital Luis Calvo Mackenna, Santiago, Chile; 7 Instituto de Ciencias e Innovación en Medicina (ICIM), Facultad de Medicina Clínica Alemana, Universidad del Desarrollo, Santiago, Chile; Universidad Autonoma de Chihuahua, MEXICO

## Abstract

Filamentous fungi are an emergent cause of severe infections in immunocompromised patients. Timely and accurate identification is crucial to initiate appropriate therapy. Traditional identification methods are time-consuming, labor-intensive, and operator-dependent. Matrix-assisted laser desorption/ionization-time-of-flight (MALDI-TOF) mass spectrometry is a rapid and easy-to-perform identification method. The effectiveness of a commercial MALDI-TOF MS platform to identify filamentous fungi in a clinical laboratory was evaluated. The study included 67 fungal isolates from 35 species/species complexes, which were identified and confirmed in mycology reference laboratories; 32 derived from clinical samples, 34 from strain collections and one was an ATCC strain. The study used the VITEK^®^ MS system (v3.2.0 database), after sample extraction by VITEK^®^ MS Mould Kit. Results were classified as “correct species”, “correct species complex”, “correct genus”and “no identification”. VITEK^®^ MS correctly identified 91.0% of isolates (58.2% to species, 29.9% to species complex, and 1.5% to genus level only). In 82%, the result matched the species/species complex identified by reference methods. No misidentifications were observed. The kit was rapid and easy to use. In conclusion, the VITEK^®^ MS system showed a high capability to accurately identify filamentous fungi in a clinical laboratory.

## Introduction

Filamentous fungi or molds are an emerging problem in clinical practice. Infections with these fungi range from mild onychomycosis to severe invasive disease in immunocompromised patients. The high mortality of systemic mold infections requires a prompt initiation of specific antifungal therapy. However, some species are intrinsically resistant to certain antifungals and susceptibility testing is not widely available. Therefore, rapid and accurate species identification is crucial for the selection of appropriate antifungal treatment [1]. This diagnostic goal, however, is often difficult to achieve and also challenged due to the description and emergence of new fungal species [2, 3].

Traditional identification techniques for filamentous fungi are based on their morphological and physiological characteristics and require experienced mycologists, a resource that is scarce in most microbiological laboratories [4, 5]. Additionally, these methods are time-consuming and not always accurate [6–8]. DNA amplification and sequencing is an additional diagnostic tool; however, this approach is primarily utilized in reference laboratories, since available kits are not approved for in vitro diagnostics [9].

Matrix-assisted laser desorption/ionization-time-of-flight (MALDI-TOF) mass spectrometry has revolutionized the diagnosis of bacteria and yeast, but its capacity to identify filamentous fungi is less studied [7]. Early reports showed poor performance, probably due to suboptimal extraction procedures and limited pathogen databases [10]. Subsequent studies demonstrated that extended databases and optimized extraction protocols can significantly improve the diagnostic capacity of MALDI-TOF [11, 12]. For example, the comparison of three pretreatment procedures applied to different MALDI-TOF instruments showed better diagnostic efficacy when cell wall disruption agents were used before analysis [13]. However, the optimization of pretreatment and extraction of mold isolates is still topic of ongoing studies [13].

In 2017, the VITEK^®^ MS 3.0 system received FDA approval for a Mould Kit (inactivation/extraction reagents] and database version, which showed promising results in initial evaluations [12]. An updated version of the database (Knowledge Base v3.2.0) was released in 2018, containing 55 additional fungal taxa. For routine laboratories, the VITEK^®^ MS system might represent a unique diagnostic alternative, if expert mycologists are not available. The present study aimed to evaluate the specificity and practicality of this new kit together with the updated database on the VITEK^®^ MS platform to identify filamentous fungi in a microbiology laboratory in Chile.

## Materials and methods

### Mold isolates

Of 67 isolates, 32 were cultured from routine clinical samples during 2017 and 2018 in hospital laboratories in Santiago, Chile: Clínica Alemana (n = 14), Hospital Clínico Red de Salud UC-CHRISTUS (n = 12), Hospital Militar de Santiago (n = 5), and Hospital Luis Calvo Mackenna (n = 1). Strains were isolated from specimens of different anatomical sites (S1 Table) and handled following standard protocols of the participating centers. Cultures were transported at room temperature in screw-cap tubes containing Sabouraud agar and in accordance to international recommendations. Further 34 isolates were from strain collections of the Mycology Laboratory, Universidad de Valparaíso (n = 28), and the Mycology Laboratory, Universidad de Chile (n = 6). These strains were of clinical or environmental origin (S1 Table). One isolate was the ATCC reference strain *Aspergillus brasiliensis* 16404.

### Reference identification

Of the 32 clinical isolates, 31 were identified in the Mycology Laboratory of the Universidad de Valparaíso and one isolate (*Coccidioides posadasii*) was confirmed by PCR and sequencing of the ITS-region in the Mycology Section, Robert-Koch Institute (Berlin, Germany), as previously reported [14] (Table 1). In the Mycology Laboratory in Valparaíso, strains were morpho-physiologically identified using dichotomous reference keys [15–17]. These included growth on specific culture media at selected temperatures, macroscopic characteristics such as growth pattern, surface and reverse colony color, texture, grooves, detection of survival and/or sexual structures, pigment production, exudates, and presence of conidial chains were part of the analysis. Microscopic examination included the measurement of the distinct structures of each species (average), hyphae, conidiophores, branches, vesicles, phialides, metulae, micro and macroconidia and the description of their shapes and surface appearance (smooth, rough, finely rough or spiny). Species identification was confirmed by molecular methods in 28 strains (Table 1) using different targets and sequencing (S1 File). The additional 34 isolates from strain collections had been identified previously by traditional and molecular methods (Table 1). Sequencing data from three of these strains were submitted to genbank [18–20].

**Table 1:** Identification 67 isolates belonging to 35 species/species complexes of filamentous fungi by VITEK^®^ MS system.

Reference identification			VITEK^®^ MS identification level			
	Method	N	Correct species	Correct species complex	Correct genus	No ID
**Hyaline molds**						
*Aspergillus brasiliensis*	NA^a^	1	1	0	0	0
*Aspergillus calidoustus*	MP/MB	2	2	0	0	0
*Aspergillus flavus*	MP/MB	2	0	2	0	0
*Aspergillus fumigatus*	MP/MB	12	12	0	0	0
*Aspergillus nidulans*	MP/MB	1	1	0	0	0
*Aspergillus niger* complex	MP/MB	6	0	6	0	0
*Aspergillus sydowii*	MP/MB	1	1	0	0	0
*Aspergillus terreus* complex	MP/MB	4	0	4	0	0
*Aspergillus tritici* ^b,c^	MP/MB	1	0	0	0	1
*Aspergillus versicolor*	MP/MB	1	1	0	0	0
*Fusarium oxysporum* complex	MP	2	0	2	0	0
*Fusarium proliferatum*	MP/MB	2	2	0	0	0
*Fusarium solani* complex	MP	3	0	3	0	0
*Penicillium brevicompactum*	MP/MB	1	0	0	0	1
*Penicillium canescens* ^b^	MP/MB	1	0	0	0	1
*Penicillium chrysogenum*	MP/MB	2	2	0	0	0
*Penicillium expansum*	MP/MB	1	0	0	0	1
*Penicillium roqueforti*	MP/MB	1	1	0	0	0
*Pseudallescheria boydii*	MP/MB	1	1	0	0	0
*Purpureocillium lilacinum*	MP/MB	1	1	0	0	0
*Sarocladium kiliense*	MP/MB	1	1	0	0	0
*Sarocladium strictum*	MP/MB	1	1	0	0	0
**Dermatophytes**						
*Epidermophyton floccosum*	MP/MB	1	1	0	0	0
*Trichophyton rubrum*	MP/MB	5	5	0	0	0
*Trichophyton tonsurans*	MP/MB	1	1	0	0	0
**Dimorphic fungi**						
*Coccidioides posadasii*	MP/MB	1	0	0	1	0
**Melanized molds**						
*Alternaria alternate*	MP/MB	1	1	0	0	0
*Curvularia spicifera*	MP/MB	1	1	0	0	0
*Sporothrix chilensis* ^b,d^	MP/MB	1	0	0	0	1
*Sporothrix globosa* ^e^	MP/MB	1	0	1	0	0
**Mucorales**						
*Lichtheimia corymbifera*	MP/MB	2	2	0	0	0
*Mucor velutinosus*	MP/MB	1	1	0	0	0
*Rhizopus arrhizus* complex	MP	1	0	1	0	0
*Rhizopus delemar* ^b^	MP/MB	1	0	0	0	1
*Rhizopus microsporus*	MP/MB	2	0	2	0	0
**Total**		**67**	**39**	**21**	**1**	**6**

ID, identification; MP, morpho-physiological identification; MB, molecular biology identification.

^a^ATCC strain N° 16404

^b^Not within VITEK^®^ MS database

^c^For further information on isolate, see reference 18

^d^For further information on isolate, see reference 19

^e^For further information on isolate, see reference 20

### Identification by VITEK^®^ MS

Isolates were subcultured on potato dextrose agar (bioMérieux, Marcy-l’Étoile, France) at 35°C or 30°C (dermatophytes) and tested by MALDI-TOF, when first growth was visible. As a first step, fungal colonies were inactivated and extracted with the VITEK^®^ MS Mould Kit (bioMérieux), which includes solutions R1 to R3, following the manufacturer´s instructions. In brief, the mycelium was collected under a biosafety cabinet with a sterile cotton swab (Classiqswabs, Copan Italia SpA, Brescia, Italy), wetted with sterile deionized water, and inoculated into 0.9mL of R1 (70% ethanol), vortexed, and centrifuged at 12,000 x g for two minutes. The supernatant was discarded, and the pellet was suspended in 40μL of R2 (70% formic acid) and vortexed. Finally, 40μL of R3 (100% acetonitrile) were added and mixed, followed by centrifugation at 12,000 x g for two minutes. One microliter of the supernatant was transferred to the target slide, dried at room temperature, and covered with 1μL of VITEK^®^ MS-CHCA matrix solution. After single extraction, isolates were tested on the VITEK^®^ MS system (bioMérieux) in a double spot manner. If no identification was achieved, the extraction and identification process was repeated. The VITEK^®^ MS software version 1.1.1 with database v3.2.0 was used for the analysis. As stated by the manufacturer, confidence values between 60.0 and 99.9 indicated a reliable discrimination to species or species complex level. *Escherichia coli* ATCC 8739 was used as a calibrator and internal control for each acquisition group, as recommended by the manufacturer.

### Classification of results

MALDI-TOF results were compared to identification obtained by the reference methods. The highest level of concordance was classified as “correct identification to species level”, “correct identification to species complex level”, “correct identification to genus level”, “incorrect identification” and “no identification”, as described in a previous study performed at Clínica Alemana [21]. Isolates from species/species complexes, which were within the database, but did not yield identification upon repeated testing, were classified as “no identification”. For species not included in the VITEK^®^ MS database, results were interpreted as “no identification”.

## Results

The study included a total of 67 isolates of filamentous fungi, belonging to 30 species and five species complexes within 15 genera. The results of individual strains can be found in the supporting information (S1 Table). With 46% of isolates, *Aspergillus* was the predominant genus. Of the 30 included species, 20 (66.7%) were correctly identified by VITEK^®^ MS; three (10%) (*Aspergillus flavus*, *Rhizopus microsporus*, and *Sporothrix globosa*) and one (*Coccidioides posadasii*) (3.3%) were correctly classified within the species complex and genus, respectively (Table 1). Six species were not identified, of which four (*Aspergillus tritici*, *Penicillium canescens*, *Rhizopus delemar*, and *Sporothrix chilensis*) were not included in the database; *Penicillium brevicompactum* and *Penicillium expansum* were not identified, albeit being part of the library database. All five species complexes were correctly diagnosed by VITEK^®^ MS (Table 1).

Of the 67 isolates, 61 (91.0%) were correctly identified by VITEK^®^ MS; 39 (58.2%) to the species level, 21 (29.9%) to the species complex level, and one (1.5%) to the genus level (Table 2). Six strains (9.0%) were not recognized by VITEK^®^ MS. Overall, in 55 isolates (82.0%; 39 species and 16 species complex) VITEK^®^ MS result coincided with the identification of the reference laboratories (Table 2). No misidentifications were observed. All VITEK^®^ MS results were unambiguous; all given identifications were provided with the maximum discrimination value (99.9%).

**Table 2:** Diagnostic performance of VITEK^®^ MS system in 67 isolates of filamentous fungi.

	VITEK^®^ MS identification level			
Reference identification	Correct species	Correct species complex	Correct genus	No ID
**Species (n = 51)**	39	5	1	6
	(76.5%)	(9.8%)	(2.0%)	(11.8%)
**Species complex (n = 16)**	0	16	0	0
		(100%)		
**Total (n = 67)**	39	21	1	6
	(58.2%)	(29.9%)	(1.5%)	(9.0%)

ID, identification

The performance of the VITEK^®^ MS system showed variations among the tested genera. The genus *Aspergillus* accounted for 31 (46.3%) of isolates, with Fumigati as the predominant section (n = 12). All *Aspergillus fumigatus* isolates were identified to the species level by VITEK^®^ MS. Other clinically important, but less frequent members of the genus *Aspergillus*, such as *Aspergillus flavus*, *Aspergillus niger*, and *Aspergillus terreus* were identified to the species complex level (Table 1). *Fusarium* was the second most frequent genus comprised in the study. All isolates of the three included *Fusarium* species or species complexes were correctly identified. Of the five *Penicillium* species, two were correctly identified; the other three were not recognized by VITEK^®^ MS, although two were present in the database. Five out of six Mucorales isolates (i.e., *Lichtheimia corymbifera*, *Mucor velutinosus*, *Rhizopus arrhizus* complex, and *Rhizopus microsporus*), were identified and only *Rhizopus delemar*, which was not included in the library, failed to be recognized.

## Discussion

The present study examined a broad spectrum of filamentous fungi, including various clinically relevant species such as *Aspergillus* spp., *Fusarium* spp. and species of Mucorales and dematiaceous fungi. Timely and correct identification of such fungal isolates is of high clinical priority, allowing a prompt adaptation of antimycotic therapy. This goal is often not achievable using traditional methods, which often require weeks until the necessary growth of mature fungal colonies [6]. Besides, the shortage of well-trained and experienced mycologists further prolongs the time to diagnosis, since difficult-to-identify isolates must be sent to reference centers [4]. Under these aspects, the reduction of time to reliably identify mold species in a routine laboratory is potentially the major advantage of commercial MALDI-TOF platforms. Since its implementation in Clínica Alemana, VITEK^®^ MS has increased the speed and accuracy of microbiological diagnosis, especially in cases of difficult-to-identify microorganisms [21, 22]. The present study showed similar results for filamentous fungi, which were processed and identified after 48–72 hours of incubation, with the initial growth of mycelia. This is in accordance with previous studies, in which identification was achieved after 48 hours [7, 8, 11]. In early reports, the use of intact fungal cells hindered mass spectrometry processing due to the rigid cell walls of molds [23]. Protocols that use an on-plate formic acid extraction method have shown insufficient results [13, 24]. However, the development of specific extraction solutions, such as the here tested VITEK^®^ MS Mould Kit, aimed to overcome this limitation [23, 25].

Another crucial factor for the identification of less frequent pathogens is the system´s database. Previous diagnostic studies of filamentous fungi using the former database version (v3.0) showed high rates of misidentifications [12, 26, 27] or identification only to the species complex level [28]. The v3.2.0 database utilized in the present study exhibited a better performance and coincided with the reference identification in over 80% of isolates. *Fusarium* spp., for example, were correctly identified in the present study (n = 7), while in studies, using the older library version, only 65%-70% were accurately diagnosed [26, 28]. Furthermore, cryptic species such as *Aspergillus sydowii* and *Aspergillus versicolor*, which pose a treatment challenge due to their azole resistance, were correctly detected [29]. Of notice, there were no misidentifications, which is of clinical importance, avoiding incorrect or sub-optimal treatment, in accordance with previous data [13].

*Aspergillus fumigatus*, grouped in the Fumigati section, is the most prevalent cause of invasive mold infection [30]. This species was the most common in the study and correctly identified in all cases. Accurate diagnosis of this species has become crucial due to the emergence of azole-resistant cryptic species within this section, such as *Aspergillus lentulus*, *Aspergillus novofumigatus*, *Aspergillus fumigatiaffinis*, *Aspergillus thermomutatus* (*Neosartorya pseudofischeri*), and *Aspergillus viridinutans* [31]. These and other members of this section associated with invasive fungal diseases were not reliably identified by the earlier version (v3.0) of the platform [31]. Those species are now included in the new database version. However, the study did not include such cryptic Fumigati species. Isolates belonging to the *Aspergillus niger* and *Aspergillus terreus* complexes were correctly identified to the complex level, as with the older library (v3.0) [28]. The v3.2.0 database now also includes some cryptic *A*. *niger* complex species such as *Aspergillus tubingensis*. The work included cryptic species of other sections (*Aspergillus calidoustus* and *Aspergillus sydowii*), which were correctly identified. *A*. *tritici* was not included in the database and, therefore, not identified.

The genus *Fusarium* is mainly described as a phytopathogenic fungi. However, *Fusarium oxysporum* complex and *Fusarium solani* complex are able to cause local (e.g., ocular) to life-threatening infections in immunocompromised patients [32]. *Fusarium solani* complex was most virulent in animal models; furthermore it exhibits amphotericin B, voriconazole, and posaconazole resistance [28, 33]. The rapid and accurate identification of this complex is therefore clinically relevant. This and previous studies demonstrated that the newer VITEK^®^ MS system exhibits a better performance than other MALDI-TOF instruments [24].

*Penicillium* spp. are of low human pathogenicity. *Penicillium chrysogenum* is the main species isolated from dwellings and considered an important cause of allergic rhinitis and asthma [34]. Of the *Penicillium* species included in this study, the VITEK® MS only identified *P*. *chrysogenum*, while *P*. *brevicompactum* (agent of hypersensitivity pneumonitis for wood workers) and *P*. *expansum* were not identified, although within the database. *Penicillium canescens* was not part of the database, so no identification was obtained. These limitations within the *Penicillium* genus have been described previously and might require further updates of the database [27].

The most common Mucorales involved in human disease are *Rhizopus*, *Mucor* and *Rhizomucor* [35]. An accurate identification of Mucorales is clinically relevant due to species-specific clinical presentations (e.g. *Rhizopus arrhizus* causing rhinocerebral invasion). VITEK^®^ MS correctly identified the included Mucorales to the species/species complex level [36]. However, only a low number of Mucorales strains were included, so further studies are warranted. This was also the case with dematiaceous fungi.

*Coccidioides posadasii*, a dimorphic fungus, was correctly identified as *Coccidioides immitis/posadasii* and signaled by the device as a biosafety risk. The capability of MALDI-TOF to identify this fungus permitted timely measures for the prevention and control of laboratory acquired infections, as reported previously [14]. This is an important add-on for laboratories in non-endemic countries, since clinical information leading to the suspicion of endemic mycosis, are often not available. Though dimorphic fungi are identifiable by VITEK^®^ MS and it is the only FDA approved database for their diagnosis, the need for an inactivation step in a biosafety level 3 (BSL-3) facility and the requirement of a minimal amount of culture biomass for identification, will probably preclude its use in a routine BSL-2 facility [37].

The laboratory personnel evaluated the Mould Kit as easy-to-use, permitting an adequate workflow integration. Since all reagents were provided as ready-to-use components, hands-on time of the inactivation/extraction steps was short (30 to 60 minutes). The smear preparation was the same as for bacteria or yeasts, including the calibration step using *Escherichia coli* ATCC 8739 strain. The simplicity of sample preparation and data analysis is an advantages of fungal identification by MALDI-TOF leading to economical savings in laboratory costs [4, 38]. Until recently, sequencing was the only alternative for unidentifiable mold strains, resulting in much higher expenses and time losses compared to MALDI-TOF [39]. A major drawback to the implementation of the VITEK^®^ MS Mould Kit in small or medium size laboratories is the package size (100 tests per box) together with an expiry period after opening of four weeks.

The MALDI Biotyper^®^ system (Bruker Daltonik GmbH, Bremen, Germany) is another MALDI-TOF MS platform frequently used in clinical laboratories. To date, few comparative data regarding the diagnosis of filamentous fungi by the Bruker and VITEK® MS systems are available. A study from France reported a superior overall identification rate of the VITEK^®^ MS, due to higher species complex level identifications [40]. In another report from the USA, the Bruker Biotyper produced fewer misidentifications of species not included in the database, reached more species level identification, but had a more complex extraction protocol [41].

Main limitations of the study were the small sample size and the incomplete spectrum of species, especially among cryptic *Aspergillus* species, Mucorales and dematiaceous molds. Additional studies are needed to confirm the results of these rare species and species complexes.

In conclusion, the study demonstrated the capability of the VITEK^®^ MS system to successfully identify a broad spectrum of filamentous fungi in a routine microbiological laboratory. Overall, the system diagnosed over 90% of the isolates without misidentifications.

## Supporting information

S1 Table Data set. Origin, sample type, identification (ID), and identification score (ID%) of included fungal isolates.
(XLSX)

S1 File Molecular identification. Molecular identification of mold isolates in the Mycology Laboratory, Universidad de Valparaíso.
(DOCX)
