# Supplementary material for: Performance of the VITEK® MS system for the identification of filamentous fungi in a microbiological laboratory in Chile
Source: PLoS One. 2024 Dec 23;19(12):e0315690. doi: 10.1371/journal.pone.0315690 (PMC11666008; doi:10.1371/journal.pone.0315690)
Supplement: S1 File — Molecular identification of mold isolates in the Mycology Laboratory, Universidad de Valparaíso. (DOCX) [file pone.0315690.s002.docx]

**Supporting information**

**Performance of the VITEK MS system for the identification of filamentous fungi in a microbiological laboratory in Chile**

**S2 File.** **Molecular identification.** Molecular identification of mold isolates in the Mycology Laboratory, Universidad de Valparaíso.

DNA extraction was performed by the CTAB protocol described by Kumar et al. (Kumar MS, Kaur G, Sandhu AK. Genomic DNA Isolation from fungi, algae, plant, bacteria and human blood using CTAB. Int J Sci Res 2012, 3: 617-8). Different genes were amplified according to the genus to be identified (see Table), with the following protocol per reaction: 12.5μl of DreamTaq M.Mix; 0.5μl of each primer (0.2μM); 5μl of DNA for a final volume of 25μl.

**Table. Targets and protocols used for different genera of molds**

| **Gen** | **Primer sequences** | **Amplification protocol** | **Genus** |
| --- | --- | --- | --- |
| ITS | ITS 1 5’-TCCGTAGGTGAACCTGCGG  ITS 4 5’-TCCTCCGCTTATTGATATGC | 95°C inicial 3 min  95°C 1 min  55.5°C 30 sec  72°C 1 min  *35 cycles  72°C final 10 min | *Penicillium*  *Pseudallesheria*  *Purpureocillium*  *Trichophyton*  *Epidermophyton*  *Curvularia*  *Alternaria*  *Sporothrix*  *Mucor*  *Rhizopus*  *Lichtheimia*  *Sarocladium* |
| Calmodulin | Cmd 5 5’- CCGAGTACAAGGAGGCCTTC  Cmd 6 5’- CCGATAGAGGTCATAACGTGG |  | *Aspergillus*  *Sporothrix* |
| Beta-tubulin | Btb2a 5’ - GGTAACCAAATCGGTGCTGCTTTC  Btb2b 5’ - ACCCTCAGTGTAGTGACCCTTGGC |  | *Aspergillus* |
| D1/D2 | NL1 5’ - GCATATCAATAAGCGGAGGAAAAG  NL4 5’ - GGTCCGTGTTTCAAGACG | 95°C inicial 3 min  95°C 30 sec  58°C 30 sec  72°C 1 min  *30 cycles  72°C final 10 min | *Sarocladium* |

For the purification step, the Wizard^®^ SV Gel and PCR Clean-Up System Protocol kit (Promega) using the gel protocol was used. The amplification products were sequenced with a 3730 xl/DNA Analyzer (Thermo Fisher Scientific).
